# The significance of mentorship in supporting the career advancement of women in the public sector

**DOI:** 10.1016/j.heliyon.2021.e07321

**Published:** 2021-06-16

**Authors:** M. Mcilongo, K. Strydom

**Affiliations:** a Business School, Nelson Mandela University, South Africa; b Faculty of Business Science, Walter Sisulu University, South Africa

**Keywords:** Career advancement, Mentorship, Previously disadvantaged, Protégé, Public sector, Women empowerment

## Abstract

Historically, patriarchy has manifested itself in the workplace and influenced career opportunities afforded to women in the public sector. The slow progress in the transformation of organisations indicates there is a need for a structural developmental approach for women's career advancement. Mentoring has been recognised as a valuable development strategy and an affirmative action tool that can be used to support and promote women and groups that have been viewed as previously disadvantaged. The aim of the study was to highlight the significance of mentorship as a career advancement mechanism for women in the South African public sector. The study identified dimensions of mentorship (female mentors, career support, mentoring policy and leadership development) and tested the proposed hypotheses to determine whether a statistically significant relationship existed between mentorship and career advancement. A quantitative approach was followed to collect data from a sample of 200 women employed in the public sector in the different provinces of South Africa. Statistical methods used to conduct the data analysis included descriptive statistics and inferential statistics. The results indicate that women employees in the public sector view mentorship as an important factor for development; however, the gender of the mentor does not necessarily influence career advancement. Mechanisms to support mentoring have not been established in the public sector, highlighting the urgency for managers in the public sector to ensure that mentorship policies are put in place.

## 1 Introduction

*“A mentor is someone who allows you to see the hope inside yourself.”* Oprah Winfrey

Women in South Africa should be “allowed’’ to see the hope within themselves and be empowered for career advancement in the workplace. Studies indicate that organisations with more females in senior and executive positions are more successful in terms of decision making, productivity and turnover (Blumberg, 2018; Onley, 2016). Yet, gender equality in the workplace is still a myth (Coetzee, 2017). Booysen and Nkomo (2014) hold a similar view, highlighting that social inequality “remains stubborn” in South Africa. It seems that women need to “climb every mountain, search high and low” (Hammerstein II, 1959) to advance in their careers. Structural inequality in the workplace results in the unequal and unfair treatment of women at work (Bosch, 2017). In addition, transformation priorities in South Africa necessitate an increase of black South African women in the workforce (Jaga et al., 2018). This study suggests that mentorship in the public sector could be a vehicle to inspire, develop and empower previously disadvantaged women in the workplace.

Mentors with advanced experience and knowledge who are devoted to contributing to the development of their protégés or mentees can make a significant difference to women who aspire to be future leaders in organisations (Samier, 2000; Satter and Russ, 2007; Shen and Kram, 2011; Weimers et al., 2013). Smith (2016) concurs and highlights that mentoring is essential to the career advancement and success of professional black African women. As women face more barriers than men in the workplace, studies suggest that females have a greater need than males to be mentored (Abalkhail and Allan, 2015; Hills, 2015; Kammeyer-Mueller and Judge, 2008; Johnson and Mathur-Helm, 2011). However, in the South African public sector, this phenomenon is not recognised and mentorship for women seems to be “non-existent” (Rankumise, 2013). Women in the public sector realise that they have to fend for themselves as they try to find their own way to survive or advance in their departments.

Policies and legislation, including South Africa's Employment Equity Act (No. 55 of 1998), the Constitution (No. 108 of 1996), the White Paper on Human Resources Management in the Public Service of 1997, and the Women Empowerment and Gender Equality Bill (Government Gazette, 2013), are in place, yet the reality in the workplace seems to contradict these policies. Twenty-seven years into the country's democracy and transformation, men still hold most decision-making positions in the public service sector (Department of Women - Annual Report, 2015/2016). Reports indicate that even though women constitute more than 60% of employees in the public sector, they tend to hold lower levels of occupational categories, namely, production and supervisory levels (DPSA Annual Report, 2015/2016). Even “good-practice diversity interventions” are not influencing and advancing women to an extent which would ensure equal and favourable conditions for women in the South African workplace (Booysen and Nkomo, 2014). Singh (2017) argues that transformation for organisations is merely about “ticking the box” and suggests organisations need to find new mechanisms to eliminate the barriers that discriminate against women. In line with this view, Bosch (2017) proposes that human resources management in organisations needs to devise policies and interventions for women in order to manifest fairness in organisations.

Government departments are continually struggling to retain, reward and develop their pool of public servants (Poocharoen and Lee, 2013). Yokwana (2014) suggests that the lack of mentorship for women results in slow career development, and a vast number of junior female employees opt to resign from their organisations due to a lack of promotion. For bureaucratic organisations in the public service sector to be filled with capable, skilled and competent employees, the sector needs to empower and advance female employees (Roger, 2010).

Career advancement and promotion generally take place after employees acquire additional skills, experience, knowledge or education. A literature review of various career theories – for example, the Career Adaptability Theory (Bocciardi et al., 2017) and Boundaryless Career Theory (Kuron et al., 2016) – reveals certain factors that influence career advancement. These career theories, as well as literature on career advancement, suggest that factors influencing the career advancement of women can be categorised as either self-driven factors or employer-driven factors (Bocciardi et al., 2017; Holton and Dent, 2016; Kuron et al., 2016). Self-driven factors include pre-career choices (for example, education), while employer-driven factors include mentorship opportunities (Böhmer and Schinnenburg, 2016; Netnou and Strydom, 2020) – supporting the view that it is important for employers to introduce mentorship initiatives to promote the career advancement of women.

Although the literature indicates positive outcomes of mentorship for the organisation and individuals, it is virtually silent on the role, outcomes and consequences of mentorship for female employees in the South African public sector. The primary objective of this study is to elevate the importance of mentorship in the culture of the South African public sector to support the advancement of junior female employees.

## 2 Literature review

The rise of women to leadership positions in South Africa is hampered by a number of barriers (Bosch, 2017; Coetzee, 2017; Mokoena, 2018). These include a history of institutional racism in South Africa, where the rights of individuals were subject to race and gender, and sociocultural theories that view women as inferior to men (Chiloane-Tsoka, 2010; Hendrickse, 2004). This historical patriarchy had a negative influence on the number of management positions offered to women in the South African public sector. When considering the slow progress of transformation in the public sector, the absence of mentors is deemed to have had the biggest impact on the career advancement of women (Arnesson and Albinsson, 2017; Hills, 2015; Mokoena, 2018; Satter and Russ, 2007; Weimers et al., 2013). There are, however, diverse views on the definition of mentorship. In the South African context, there is disagreement on the meaning of the term “mentorship” when comparing it to activities that are similar, such as coaching and counselling (Goosen, 2009).

Mentoring in business studies finds its roots in the early work of Kram (1985, p. 23), in which mentoring is described as “developmental relationships between younger and older managers that promote individual development through career stages”. Lankau and Scandura (2002), on the other hand, describe mentoring as a relationship that contributes to personal growth without restricting the relationship to seniority and power. Mentorship is commonly professed to be a dynamic and transformative relationship which affects both parties – the mentor and mentee, or protégé – as well as their respective careers and personal and professional development (Arnesson and Albinsson, 2017; Cooke et al., 2017; Roger, 2010). Other elements mentioned in the literature as essential to the mentoring relationship include that it is a relationship built on trust; it should possess a domain of ethics; and the mentor should be a role model to the mentee (Bozeman and Feeney, 2009; Harris and Leberman, 2012; Jyoti and Sharma, 2015).

In clarifying the term “mentorship”, Arnesson and Albinsson (2017) refer to the misperception regarding mentorship and supervision, specifically the similarities and differences between these two concepts. They explain that supervision is *compulsory* and includes *evaluation*, whereas these obligations do not apply to mentorship. The advantages of a mentoring relationship, as depicted in these descriptions, include improved job performance and job satisfaction (Cooke et al., 2017). In summarising the various definitions of mentorship, it is noted that all contain a common thread in that they involve an exchange of benefits between the mentor, the protégé and the organisation.

Western and Asian countries have provided much literature on the significance of mentorship in the success of women's careers; however, most of the studies are focused on a single country or a single sector (Abalkhail and Allan, 2015; Arifeen, 2010; Mustafa et al., 2014; Yokwana, 2014). Therefore, transferability to South Africa's public service context is unclear. For example, Bhatta and Washington (2003) state that in the Japanese public sector, junior staff members are assigned to a mentor when they join the public service sector, and the mentor guides them for a considerable period of time, while in the elite public service sector of Singapore, junior staff members are assigned to a mentor who offers them friendly advice and training. Furthermore, Abalkhail and Allan (2015) provide contrasting views regarding the importance of mentorship to women in junior positions in two countries, namely, Saudi Arabia and the United Kingdom (UK). Their study concludes that women in Saudi Arabia do not recognise the importance of mentorship in the workplace, mainly due to cultural beliefs, as they consider mentors as only members of their family. However, women in the UK view mentorship as important to their career development.

Regrettably, such mentorship opportunities are not prevalent in the public sector of South Africa. Women in the South African public sector are therefore compelled to develop themselves and climb the hierarchical ladder without being mentored (Coetzee, 2017; Weimers et al., 2013). A further challenge is that the public sector is rigid, with inflexible rules, and this type of organisational structure could hamper the advancement of women (Sadangharn, 2010). Literature suggests that, for a mentoring culture to exist, management needs to be flexible and progressive so strategies can be adopted and policies revised (Apospori et al., 2006; Jyoti and Sharma, 2015; Mokoena, 2018). In addition, the public sector in South Africa needs to consider the outcomes of the Women Empowerment and Gender Equality Bill (Government Gazette, 2013), which include “equal representation and an increased participation by building females’ capacity”. This Bill provides further justification for exploring the significance of mentorship in supporting the career advancement of women in the public sector.

## 3 Theoretical framework

The framework guiding this study was informed by existing theories and models of mentorship found in the literature. Table 1 below provides the justification for the dimensions of mentorship as applied and tested in this study.

**Table 1:** Mentorship dimensions with supporting theories or models.

MENTORSHIP DIMENSION	THEORY OR MODEL
Mentoring support for female employees by female mentors	The similarity attraction theory of mentorship states that people have a tendency to be attracted to people they deem similar to them (Kalbfleisch and Davies, 1991; Young et al., 2006). When women find role models and mentors in other women, their productivity and careers can be enhanced. This theory also suggests that female employees are more likely to understand female managers than male managers (Valentine and Godkin, 2000; Mello and Phago, 2007).
Mentoring career support (coaching, networking) can assist women in career advancement	The Clutterbuck empirical model for mentorship views mentorship as a way to expand knowledge and skills through coaching, guiding, counselling and networking (Stroude et al., 2015). Career support that includes coaching, sponsoring protégés and providing challenging assignments is argued to help protégés advance along the hierarchical ladder of the organisation (Tharenou, 2005).
Mentoring policy for women can assist in career advancement	Zey's (1989) model for mentorship (as quoted in Samier, 2000, p. 89) advocates for a mentoring policy to be developed in public sector organisations. Such a policy can lead to formal programmes, and it has been reported that formal mentorship programmes can lead to career advancement (Ehrich, 2008).
Mentoring for leadership development of women	Mentorship plays a significant role in leadership development and continuity in the public sector (Bozeman and Feeney, 2009). Bozeman and Feeney's (2009) three-tier model for mentoring in the public sector maintains that it is very important for the public sector to groom its leadership. This model therefore views mentorship as a tool that can be used to prepare executives and to achieve adaptation.

Source: Authors' own construction.

### 3.1 Proposed framework for the hypothesised relationships

The theories and models in Table 1 provided the framework for the mentorship dimensions and the proposed hypotheses.

• **Female mentors**

Coetzee (2017) proposes that organisations should cater specifically for the unique needs of women. Studies suggest that female mentors are best for the career development of other females (Arifeen, 2010; Mello and Phago, 2007; Tharenou, 2005). Gerber and Schlechter (2019) found that women embrace solidarity and support each other. Women who have female mentors can learn more strategies for dealing with barriers in the workplace and gain more career support than if they had a male mentor (Tannen, 2010). In addition, “black African women in leadership serve as important role models to a younger generation of South African women” (Bosch, 2020).

The following hypothesis was therefore proposed:

H1 There is a positive relationship between female mentor support and the career advancement of junior female protégés.

• **Career support**

Mentor career support involves sponsoring the protégé in order to advance them through coaching, giving challenging assignments, providing network connections, and making protégés visible in the organisation (Eby et al., 2004). It can therefore be argued that mentor career support can ensure that a protégé advances in the hierarchy of the organisation. Pan et al. (2011) concur with the notion that mentor career support can help women advance and highlight that it is a very important element of mentoring. Madsen and Andrade (2018) propose that training and development, and thus also mentoring, should address unconscious gender bias at the workplace. This led to the following hypothesis being proposed:

H2 There is a positive relationship between mentor career support and higher levels of career advancement for junior female employee protégés.

• **Mentoring policy**

One of the reasons mentoring is advocated as a policy mechanism is that, traditionally, mentoring is selective (Ehrich, 2008; Alkadry and Tower, 2011). It is not available to all those who need a mentoring relationship. Most mentors tend to gravitate towards those protégés who they feel they have something in common with (Apospori, et al., 2006). It is for these reasons that a formal mentoring policy for females is advocated so that all potential protégés can be included. Bangura and Thomas (2015) affirm that there is a direct relationship between governance and gender equality, indicating the importance of a formal mentoring policy.

The following was therefore hypothesised:

H3 A formal mentoring policy for females in the public sector is positively related to higher levels of career advancement.

• **Leadership development**

Mmope (2020) suggests that management should consider mentorship strategies for professional black women through *ubuntu*-infused leadership. This leadership style would encourage the development of female employees. The leadership development of employees can positively affect organisational capacity and capacitate employees to assume leadership roles in the organisation (Bhatta and Washington, 2003; Potgieter, 2011; Weimers et al., 2013). Women should be empowered to be insightful, visionary leaders who create environments in which diversity and excellence thrive (Nhamo and Mjimba, 2020). Mentoring can thus enable the leadership development of employees. The following hypothesis was therefore proposed:

H4 Mentoring for leadership development has a positive relationship to the career advancement of junior female employees.

These four hypotheses were empirically tested to determine whether a statistically significantly relationship existed between mentorship and career advancement within the public sector of South Africa.

## 4 Research methodology

### 4.1 Research paradigm

A quantitative research approach was adopted for this study. The quantitative research paradigm was selected because the study involved a large number of subjects, and a quantitative approach would allow the data to be analysed numerically. Futhermore, the study presented hypotheses, and the most accurate way to test hyphotheses is with the use of the quantitative paradigm.

### 4.2 Sample

The target population for this study comprised entry-level junior females in the South African public sector, on salary levels 5 to 10 (production and supervisor-level female employees), who would benefit from mentorship and career advancement and who aspire to move up the career ladder. The South African public service workforce comprises approximately 1 244 852 people, of which 580 223 are women employed at a salary level of 5–10 (DPSA Annual Report, 2015/2016). Targeted random sampling was used for this study to ensure an unbiased sample within the target group (female employees on salary level 5 to 10), as an unbiased sample is the most appropriate criterion to evaluate the adequacy of a sample (Bryman and Bell, 2015). In addition, random sampling is representative of the entire population and enables accuracy of representation for large populations (Maree, 2016). An invitation to participate in the study was emailed to female employees in the public sector who are at salary levels 5 to 10. The decision to participate in the study was solely up to the respondents and everyone who responded positively was provided a questionnaire via email.

The formula to determine the appropriate sample size is as follows: N/(N∗(d)^2^ + 1), where N = number of population size and d = degree of confidence (Israel, 2013). The sample size for this study which yielded a 95% degree of confidence was 400. However, for the purpose of this study, it was envisaged that a sample size of 200 would be appropriate and produce statistically significant results. It is estimated that a sample size of 200 has a margin of error of 7.1%, which is acceptable, and can produce statistically significant results (Niles, 2006). Conroy (2012) also concludes that an acceptable margin of error lies between 4 and 8%, which supports the notion that the results of the study would yield acceptable results with a sample size of 200 respondents.

### 4.3 Research instrument

A structured questionnaire was drafted based information obtained from the literature. A quantitative research approach was employed to seek statistical associations and relationships between factors affecting mentorship (dependent variables) and career advancement (independent variable). Mentorship dimensions (female mentors, career support, mentoring policy and leadership development) were identified from theoretical models and were supported by literature. Hypotheses were constructed to show each factor and its relationship to career advancement. The purpose of the measuring instrument was to obtain primary data to test the hypothesised relationships.

The items on the questionnaire were anchored by a five-point Likert scale. A pilot study was conducted to test the questionnaire on five individuals who represented the target population. Further amendments, as suggested by these individuals, were made to the questionnaire. Ethics approval was received from the Research Director at Nelson Mandela University before the researchers emailed the questionnaires to the 200 female respondents.

Both content and construct validity were ensured by linking the mentoring theory and literature underlying the research to the items in the questionnaire, and by confirming that the measurements on the scales correlated with other propositions. Internal consistency was tested using Cronbach's alpha coefficient analysis, the most appropriate reliability testing method for this study. The coefficient analysis describes the extent to which all items in a test measure the same construct and is suitable for the purpose (Taber, 2017). If the items are strongly correlated with each other, their internal consistency is high and the alpha coefficient will be close to one (Bryman and Bell, 2015).

Section A of the questionnaire focused on the demographic profiles of the respondents, and Section B comprised attitude scales to determine the perceptions of respondents on the influence of mentorship on career advancement. The results of the coefficient analysis indicated that the Cronbach's alpha for Section B of the questionnaire (Mentorship), which consisted of 25 questionnaire items, was 0.840 (84%). This suggests that the questions had a relatively high internal consistency, since an acceptable reliability scale is 70% and values lower than 0.60 are regarded as unacceptable (Maree, 2016). In support of the high internal consistency of this study, Taber (2017) suggests that values of 0.80 or above indicate excellent reliability.

### 4.4 Data analysis

A questionnaire was used to obtain data from respondents; thereafter, the collected data were exported to Microsoft Excel for coding and to tabulate the data. The study employed descriptive and inferential statistics, the two most commonly used data analysis techniques for quantitative data (Bryman and Bell, 2015). Descriptive statistics were used to summarise the data and compare the variables numerically, as per Maree (2016). The mean and standard deviation of each of the statements of the mentorship dimensions, namely, mentor gender, career support, mentoring policy and leadership development, were reported.

Inferential statistics were used to generalise the findings from the sample (200 women in the public sector) to the entire population. Inferential statistics, namely, ANOVA and the Chi-square test, were employed to determine the statistical significant relationships among variables – in this case, the relationship of each mentorship dimension with career advancement – and confirm whether a hypothesis had been supported or rejected (Conroy, 2012; Taber, 2017). Concurrently, inferential statistics were used to draw conclusions from the data (Bryman and Bell, 2015). The computer software package, SPSS version 21.0., was used to execute the quantitative statistical analysis.

## 5 Results

The results included the demographic profiles of the respondents, descriptive statistics, analysis of variance (ANOVA) and chi-square. The sample size was 200, and all questionnaires that were sent out were returned unspoilt (100% response rate). Prior to conducting a detailed analysis of the data, the data were checked for normality. The normality of data is assumed when “the significance level is greater than 0.05” (Pallant, 2007, p. 54). In terms of the result, as obtained from the Kolmogorov-Smirnov (K–S) test for normality, the data were normally distributed since the p-value was 0.614.

### 5.1 Demographic profiles of the respondents

Section A of the questionnaire aimed to profile respondents based on demographic information. The aim was to establish the distribution of demographic information in terms of the respondents’ highest level of qualification and employment level/grade.

• **Highest level of education**

Career advancement is directly related to the education of candidates. This analysis therefore sought to ascertain the distribution of the respondents based on their highest level of education. In terms of the data presented, 64.5% of the respondents had acquired at least a National Diploma while 34.5% were in possession of a Senior Certificate as their highest qualification. In essence, all the respondents had acquired at least a Senior Certificate. This implies that all female employees who participated in the survey have the potential to develop if education was used as a measure of the intention to further develop their career.

• **Post level/position grades**

The purpose of this question was to identify the variations and distribution of respondents with regard to their post level or position grade. The distribution indicated that most respondents (76%) were split equally between post level 7–8 (38%) and post level 9–10 (38%). More than three-quarters of the entire sample had advanced from one level to another. Only 24% of the respondents were at level 5–6. Post level is a measure of whether an individual has advanced in terms of their career or not. This information is crucial, since the perception of people regarding the influence of mentorship on career advancement can relate to whether the individual has experienced an upgrade of their post level.

### 5.2 Analysis of variance (ANOVA)

ANOVA was conducted in order to compare differences within groups of demographic variables (employment level, highest qualification) with regard to the respondents’ knowledge of and experience with the impact of mentorship on career advancement. The Pearson correlation p-value of (p < 0.05) was used to test the significance of the differences. If the p-value is less than 0.05, it can be assumed that there is a statistically significant difference between groups or categories of the demographic variables with regard to the factor of comparison (Maree, 2016). The F-statistic states the spread or strength of the demographic in relation to factors that influence mentorship and career advancement. The higher the F-value, the larger the spread (Hurlbert, 1984).

It can be observed that the significance levels (p-values) of the determinants of mentorship are all larger than 0.05 (Table 2). The results show that the responses are similar irrespective of employment level or highest qualification, and indicate that generally the experience or opinions of the respondents did not vary much based on their employment level and education. The F-values indicates that the spread of responses varied slightly among respondents with different levels of employment and qualifications.

**Table 2:** ANOVA: Employment level and highest qualification.

Mentorship variables	Employment level		Highest qualification	
	F-value	p-value	F-value	p-value
Career support	2.058	0.130	1.384	0.241
Leadership development	0.232	0.793	0.428	0.788
Mentoring policy	0.935	0.394	0.982	0.418
Mentor gender	1.508	0.224	1.727	0.146

### 5.3 Descriptive statistics

The following section presents the results of the analysis of descriptive statistics for all the dimensions deemed to determine the influence of mentorship towards career advancement.

The results presented in Table 3 show that the dimension “Mentor gender”, with the statement “Mentoring support is more important for women's career advancement” (mean 3.61; standard deviation 1.084) and the variable “A female mentor can teach me how to deal with barriers in my organisation” (mean 3.28; standard deviation 1.023), were the most dominant factors. The factor “Mentorship is more effective if the mentor is the same gender” was the least dominant factor, with a mean of 2.66 and standard deviation of 1.274. Although all the factors suggest that the gender of the mentor plays a significant role in influencing individual career advancement, the mean values were only marginally above average. In essence, all the factors endorsed the significance of mentor gender in influencing career advancement, but inferential statistics with the help of a chi-square test provided true statistical confirmation.

**Table 3:** Descriptive statistics of the study.

Statement	Percentage distribution					Statistics	
	SD	D	N	A	SA	Mean	Std. dev.
**Descriptive statistics: Mentor gender**							
Mentorship support is more important for women's career advancement.	6.50%	7.00%	26.00%	40.50%	20.00%	3.61	1.084
A female mentor can teach me how to deal with barriers in my organisation.	6.00%	15.50%	31.50%	38.50%	8.50%	3.28	1.023
A female mentor will have the ability to influence my career advancement.	11.00%	18.00%	40.50%	23.00%	7.50%	2.98	1.075
I would prefer my mentor to be the same gender.	26.00%	17.50%	23.00%	24.50%	9.00%	2.73	1.325
Mentorship is more effective if the mentor is the same gender.	25.50%	19.50%	27.00%	20.00%	8.00%	2.66	1.274
**Descriptive statistics: Career support**							
Mentoring will increase women's career advancement.	1.50%	3.00%	10.00%	33.00%	52.50%	4.32	.884
Mentorship can provide opportunities for me to interact in a meaningful way with mentors.	1.00%	2.50%	6.50%	46.00%	44.00%	4.30	.782
Mentorship can have a positive impact on my development.	1.00%	3.50%	8.50%	39.00%	48.00%	4.29	.844
Mentoring will improve my visibility in the organisation.	1.50%	2.50%	11.50%	41.00%	43.50%	4.22	.859
Mentoring will enable me to move to the next job level.	5.50%	8.00%	16.00%	30.00%	40.50%	3.92	1.175
**Descriptive statistics: Mentoring policy**							
A mentoring policy will encourage the career advancement of women.	3.50%	3.00%	16.50%	35.00%	42.00%	4.09	1.008
A formal mentoring programme will encourage potential protégés.	1.00%	3.00%	19.00%	51.00%	26.00%	3.98	.814
A mentoring policy will encourage potential mentors.	3.00%	0.00%	20.00%	56.50%	20.50%	3.92	.819
There is a clear policy for mentoring women in my organisation.	13.50%	29.50%	27.50%	21.00%	8.50%	2.82	1.165
There is a formal mentoring programme in my organisation.	20.50%	28.50%	23.00%	23.50%	4.50%	2.63	1.179
**Descriptive statistics: Leadership development**							
Mentorship can improve leadership development in my organisation.	1.50%	2.50%	13.00%	32.00%	51.00%	4.28	.893
Mentorship can enable me to be an effective leader in my organisation.	2.00%	4.50%	7.50%	43.50%	42.50%	4.20	.908
It would be good for me to be in a position where I can develop, manage, and coordinate policies and activities.	1.50%	7.00%	12.00%	47.50%	32.00%	4.02	.927
Mentorship can help me advance to a position with more responsibility.	3.00%	4.00%	16.00%	46.50%	30.50%	3.98	.948
There is a clear strategy for leadership development of women in my organisation.	10.00%	28.50%	26.50%	24.00%	11.00%	2.97	1.171

The dimension “Career support” (Table 3) had the highest mean (4.32), suggesting that the majority of the respondents (85.5%) acknowledged that mentorship has a significant impact on women's career advancement. The second most important factor, “Mentorship can provide opportunities for me to interact in a meaningful way with mentors”, had a mean score of 4.30 and standard deviation of 0.782, which suggests that 90% of women were of the belief that mentorship provides room for interaction with mentors. Although the variable “Mentoring will enable me to move to the next job level” had the lowest mean (3.92), it was above the midpoint (2.5) and the majority of the respondents (40.5% strongly agree and 30% agree) therefore acknowledged that mentorship improved the chances of advancing to a better working position in an organisation.

According to the evidence presented for the dimension “Mentoring policy” (Table 3), approximately 77% of the respondents argued in favour of the statement, “A mentoring policy will encourage the career advancement of women”. This variable had the highest mean (4.09) and standard deviation (1.008). Furthermore, over 77 (26% strongly agree and 51% agree) of the respondents were of the opinion that a formal mentoring programme would encourage potential protégés. Although the variable “There is a formal mentoring programme in my organisation” had the lowest mean score (2.63), it was well above the midpoint (2.5). This confirmed there is a general consensus that mentoring policy plays a crucial role in supporting mentorship within an organisation, thus positively impacting the career advancement of women.

The evidence presented in Table 3 under “Leadership development” shows that the variable “Mentorship can improve leadership development in my organisation” had the highest mean (4.28), followed by “Mentorship can enable me to be an effective leader in my organisation”, with a mean of 4.20. The variable with the lowest score was “There is a clear strategy for leadership development of women in my organisation”, with a mean of 2.97. All the variables had a mean score of more than 2.5, suggesting that mentorship significantly influences leadership development, which in turn has a positive influence on the career advancement of women.

### 5.4 Hypotheses testing: Chi-square test

This study utilised Pearson's chi-square test of association in order to determine the relationships between determinants of mentorship and career advancement. Through Pearson's chi-square test, the null hypothesis is either true or false (Greenwood and Nikulin, 1996). If the value of the test is too small, then the null hypothesis is false. The linear-by-linear association is also presented to test for association among the variables. If the sig. “p” value is <0.05, it means that there is a statistically significant relationship between the variables, and the null hypothesis is rejected. However, if the sig. “p” value is >0.05, it means that there is no statistically significant relationship between the variables, which means the null hypothesis is not false, but rather the alternative hypothesis is not true (Greenwood and Nikulin, 1996).

In order to determine the association between the knowledge and experience of the participants regarding the importance of each mentorship variable and career advancement, cross-tabulation was conducted. The cross-tabulation included the following variables from the research hypotheses: female mentor support, career support, mentoring policy and mentoring for leadership development. In order to confirm the significance and strength of the relationship, the symmetric measure from the chi-square analysis was considered.

• **Hypothesis 1: Female mentor support and career advancement**

The cross-tabulation of the factors for female mentor support and career advancement was conducted and yielded the results presented in Table 4.

**Table 4:** Chi-square results: Female mentor support and career advancement.

Statistical tests	Value	df	Asymp. Sig. (2-sided)
Pearson's chi-square	18.297	16	.307
Likelihood ratio	20.977	16	.179
Linear-by-linear association	.003	1	.957
N of valid cases	200		

Assuming that Pearson's chi-square is significant at p < 0.05, Table 4 shows that Pearson's chi-square is p = 0.307, at pχ(1) = 18.297. The p-value “sig.” is above 0.05; therefore, the results suggest that there is no statistical significant relationship between female mentor support and career advancement. The result indicates that the gender of a mentor does not necessarily lead to career advancement. It can be concluded that there is no statistical significant relationship between female mentor support and career advancement; thus, the null hypothesis is not rejected: (*H*_*1*_ not accepted):

H1 Female mentor support will have a positive impact on the career advancement of junior female protégés.

• **Hypothesis 2: Career support and career advancement**

The cross-tabulation of the factors for career support and career advancement was conducted and yielded the results presented in Table 5.

**Table 5:** Chi-square results: Career support and career advancement.

Statistical tests	Value	df	Asymp. Sig. (2-sided)
Pearson's chi-square	490.009	16	.000
Likelihood ratio	313.317	16	.000
Linear-by-linear association	162.087	1	.000
N of valid cases	200

The results in Table 5 indicate that there is a statistically significant association between career support and career advancement. Pearson's chi-square is significant at p < 0.05. It can be observed that Pearson's chi-square is p = 0.000, at pχ(1) = 490.009. This indicates that career support plays an important role in supporting career advancement. In order to confirm the significance and strength of the relationship, the symmetric measures from the chi-square analysis were considered. The results indicate that there is a strong positive relationship between career support and career advancement. Therefore, the null hypothesis is rejected and the alternative hypothesis is retained, which is as follows:

H2 There is a positive relationship between mentor career support and higher levels of career advancement for junior female employee protégés.

• **Hypothesis 3: Mentoring policy and career advancement**

The cross-tabulation of the factors for mentoring policy and career advancement was conducted and yielded the results presented in Table 6.

**Table 6:** Chi-square results: Mentoring policy and career advancement.

Statistical tests	Value	df	Asymp. sig. (2-sided)
Pearson's chi-square	70.585	16	.000
Likelihood ratio	73.306	16	.000
Linear-by-linear association	32.492	1	.000
N of valid cases	200

The results in Table 6 show that there is a statistically significant relationship between mentoring policy and career advancement. Pearson's chi-square is significant at p < 0.05. It can be observed that Pearson's chi-square is p = 0.000, at pχ(1) = 70.585. The symmetric measures from the chi-square analysis were considered and the results confirm that there is a statistically significant relationship between mentoring policy and career advancement. Therefore, the null hypothesis is rejected and the alternative hypothesis is retained, which is as follows:

H3 A formal mentoring policy for females in the public sector is positively related to higher levels of career advancement.

• **Hypothesis 4: Leadership development and career advancement**

The cross-tabulation of the factors for leadership development and career advancement was conducted and yielded the results presented in Table 7.

**Table 7:** Chi-square results: Leadership development and career advancement.

Statistical tests	Value	df	Asymp. sig. (2-sided)
Pearson's chi-square	162.546	16	.000
Likelihood ratio	118.464	16	.000
Linear-by-linear association	73.865	1	.000
N of valid cases	200

According to Table 7, there is a statistically significant relationship between leadership development and career advancement. Pearson's chi-square is significant at p < 0.05. It can be observed that Pearson's chi-square is p = 0.000, at pχ(1) = 162.546. The symmetric measures in the above table confirm the significance and strength of the relationship. It can be concluded that there is a statistically significant association between leadership development and career advancement. The null hypothesis is therefore rejected and the alternative hypothesis is retained, which is as follows:

H4 Mentoring for leadership development has a positive relationship to the career advancement of junior female employees.

The results of the chi-square test indicate that three of the four dimensions of mentorship, namely career support, mentoring policy and leadership development, have a statistically significant relationship with career advancement. Contrary to the literature (Arifeen, 2010; Mello and Phago, 2007; Tannen, 2010; Tharenou, 2005), the study found no statistically significant relationship between female mentor support and career advancement.

## 6 Discussion of findings

The respondents, independent of their qualifications and employment level, did not prefer or display any peculiar affinity for the gender of the mentor, as long as they could learn from the mentor. This indicates that the gender of the mentor is not important. This is confirmed by the results of testing Hypothesis 1 (Table 4), which yielded a value of p = 0.307, indicating that there is no statistically significant relationship between female mentor support and career advancement. However, contrary to this finding, previous studies suggest that career development is improved when women find role models and mentors in other women, who are more likely to understand them (Arifeen, 2010; Gerber and Schlechter, 2019; Tannen, 2010; Valentine and Godkin, 2000).

The results also showed that the respondents felt optimistic about their career advancement through career support. The majority of the respondents (85.5%) acknowledged that mentoring career support has a significant impact on women's career advancement. This opinion of the respondents is confirmed by the results of Hypothesis 2 (Table 5), which indicate that there is a strong positive relationship between career support and career advancement. This is consistent with the literature (Madsen and Andrade, 2018; Pan et al., 2011; Stroude et al., 2015; Tharenou, 2005), which is unambiguous about the assistance of career support in advancing women on the hierarchical ladder.

The respondents, independent of their qualifications and employment level, were moderately satisfied with the role of a mentoring policy for women in the public sector and how it could positively impact their career advancement; however, the presence of a mentoring policy does not necessarily guarantee career advancement. The study was able to provide empirical evidence to support Hypothesis 3 (Table 6), concluding that there is a statistically significant relationship between mentoring policy and the career advancement of women in the public sector. This finding supports literature which reports that formal mentorship programmes can lead to career advancement, citing a direct relationship between governance and gender equality (Alkadry and Tower, 2011; Bangura and Thomas, 2015; Ehrich, 2008).

The descriptive statistics revealed that there is a significant and strong relationship between leadership development and career advancement. The majority of respondents signified that mentorship can improve leadership development for women in the public sector. It can thus be reasoned that there is a strong association between leadership development, career advancement and mentorship. This relationship is confirmed by the results of Hypothesis 4 (Table 7), which indicate a statistically significant association between leadership development and career advancement. Literature supports this finding, highlighting the significance of leadership development for women in the public sector (Bozeman and Feeney, 2009; Mmope, 2020; Potgieter, 2011; Weimers et al., 2013).

## 7 Managerial implications

In order to promote fairness and eliminate elements of patriarchy from government organisations, the government needs to devise policies, training and development for women. The government may use mentorship as an instrument to remove certain barriers that interfere with the progress of women.

Besides certain policies and legislation, including South Africa's Employment Equity Act (No. 55 of 1998), the Constitution (No. 108 of 1996), the White Paper on Human Resources Management in the Public Service of 1997, and the Women Empowerment and Gender Equality Bill (Government Gazette, 2013), the public sector does not have mechanisms in place for mentoring initiatives that support current legislation and policies. Management should ensure that such mechanisms are implemented.

Meaningful mentorship training should be provided for management to equip managers or senior employees with the necessary skills to mentor young women. In addition, training will also assist managers to identify possible protégés in their respective departments and sections. This mentorship training could be done by designing an induction plan for managers, which would ensure that managers are well equipped to mentor and assist less-experienced managers.

It is crucial that managers in the public sector recognise that mentoring can be an efficient career advancement strategy for women, with positive outcomes for both employees and the organisation. Without management's support and encouragement to ensure that mentorship practices and policies are in place and effectively monitored, these proposals will be merely suggestions on paper or strategic plans that are not operationalised and implemented.

## 8 Recommendations

Women in the public sector believe that mentorship plays an important role in their career development; however, women are not always convinced that the mentorship programme will be effectively implemented. The following recommendations are proposed:

• Management should ensure that a formal mentorship programme is developed and included in the organisation's policy for the empowerment of women in the public sector.
• Operational plans should include regular follow-ups to ensure that departments are implementing the mentorship programme through their respective Human Resources Departments.
• Management should ensure that there is a policy document that explains the process of mentorship and how the mentoring relationship is approached, and clarifies the roles of the mentor and mentee.
• A dedicated person or people in the Human Resources Department are tasked to act as the liaison between management and employees regarding the mentorship initiative.
• Public departments should engage the assistance of the private sector through consulting agencies that specialise in mentorship and coaching to assist in creating a mentorship programme that will fit the public sector and that will specifically benefit women in the public sector.

## 9 Conclusion

The aim of the study was to determine the significance of mentorship in the career advancement of women in the South African public sector. The results of the study show that junior female employees in the public sector view mentorship as an important factor in their career advancement. The results suggest that the respondents were of the opinion that the gender of a mentor does not necessarily lead to career advancement.

Even though legislation is in place to ensure equal rights and equal opportunities for women in South Africa, it is evident that mechanisms to enforce this are not in place. The findings revealed that departments in the public sector do not have formal policies for mentorship or mentorship programmes. Mechanisms to support mentoring have not yet been established.

If the recommendations of Erwee (1992), in the paper “Organizational variables influencing female advancement in South Africa” are considered, it seems that the same recommendations for women remain relevant now, 29 years later, namely: “solutions to the slower advancement of women in organizations should be sought in both personal action plans formulated by career-oriented women and creation of enabling conditions in companies”. This study suggests that it is now the time for action, and women should take a leading role in building networks and ensuring that management promotes mentorship for women as a top priority in their organisations.

Women in the public sector should not wait indefinitely for formal mentoring programmes from management. In the interim, they should take the initiative to arrange mentorship programmes and networks amongst themselves to ensure that both mentor and protégé are motivated by this developmental relationship.

*“Don't wait for someone to take you under their wing. Find a good wing and climb up underneath it.”* Frank Bucaro

## Declarations

### Author contribution statement

Kariena Strydom: Analyzed and interpreted the data; Wrote the paper.

Mihlali Mcilongo: Conceived and designed the experiments; Performed the experiments; Analyzed and interpreted the data.

### Funding statement

This research did not receive any specific grant from funding agencies in the public, commercial, or not-for-profit sectors.

### Data availability statement

Data will be made available on request.

### Declaration of interests statement

The authors declare no conflict of interest.

### Additional information

No additional information is available for this paper.
